# A Single-Side Micromachined MPa-Scale High-Temperature Pressure Sensor

**DOI:** 10.3390/mi14050981

**Published:** 2023-04-29

**Authors:** Peng Li, Wei Li, Changnan Chen, Sheng Wu, Pichao Pan, Ke Sun, Min Liu, Jiachou Wang, Xinxin Li

**Affiliations:** 1 State Key Laboratory of ASIC and System, School of Microelectronics, Fudan University, Shanghai 200433, China; 19112020040@fudan.edu.cn; 2 State Key Laboratory of Transducer Technology, Shanghai Institute of Microsystem and Information Technology, Chinese Academy of Sciences, Shanghai 200050, China; weili@mail.sim.ac.cn (W.L.); ccn@mail.sim.ac.cn (C.C.); wusheng@shanghaitech.edu.cn (S.W.); panpichao@mail.sim.ac.cn (P.P.); sunke@mail.sim.ac.cn (K.S.); liumin@mail.sim.ac.cn (M.L.); 3 School of Microelectronics, University of Chinese Academy of Sciences, Beijing 100049, China

**Keywords:** piezoresistive pressure sensor, high temperature, ultra-small size, (100)/(111) hybrid SOI wafer, front-side bulk-micromachining process

## Abstract

This paper proposes a piezoresistive high-temperature absolute pressure sensor based on (100)/(111) hybrid SOI (silicon-on-insulator) silicon wafers, where the active layer is (100) silicon and the handle layer is (111) silicon. The 1.5 MPa ranged sensor chips are designed with the size as tiny as 0.5 × 0.5 mm, and the chips are fabricated only from the front side of the wafer for simple, high-yield and low-cost batch production. Herein, the (100) active layer is specifically used to form high-performance piezoresistors for high-temperature pressure sensing, while the (111) handle layer is used to single-side construct the pressure-sensing diaphragm and the pressure-reference cavity beneath the diaphragm. Benefitting from front-sided shallow dry etching and self-stop lateral wet etching inside the (111)-silicon substrate, the thickness of the pressure-sensing diaphragm is uniform and controllable, and the pressure-reference cavity is embedded into the handle layer of (111) silicon. Without the conventionally used double-sided etching, wafer bonding and cavity-SOI manufacturing, a very small sensor chip size of 0.5 × 0.5 mm is achieved. The measured performance of the 1.5 MPa ranged pressure sensor exhibits a full-scale output of approximately 59.55 mV/1500 kPa/3.3 VDC in room temperature and a high overall accuracy (combined with hysteresis, non-linearity and repeatability) of 0.17%FS within the temperature range of −55 °C to 350 °C. In addition, the thermal hysteresis is also evaluated as approximately 0.15%FS at 350 °C. The tiny-sized high temperature pressure sensors are promising in various industrial automatic control applications and wind tunnel testing systems.

## 1. Introduction

Since the discovery of the piezoresistive effect in doped silicon [1], it has been widely used in various sensors such as gyroscopes [2], accelerometers [3] and pressure sensors [4]. Many more sensing principles have been discovered and developed in recent years. Bock W. J. put forward a free active element bulk-modulus high-pressure transducer, which is based on a fiber-optic displacement sensor. At pressure ranges up to 100 MPa, the accuracy of the transducer was estimated at 1.5% of full scale [5]. Morten B. developed a resonant pressure sensor based on the piezoelectric properties of ferroelectric thick films. When the sensor was working at a frequency of approximately 57.8 kHz, consistent with the third-mode frequency $f_{0.3}$, a shift $\Delta f_{0.3}$ = 650 Hz was found by changing the gas pressure from 0 to 900 mmHg [6]. Vasileios M. fabricated a capacitive pressure sensor for low-pressure measurement ranges. The pressure sensor performed a 0.25 kPa pressure resolution and a 10 pF capacitive change under a 6.5 kPa compressive load [7]. However, piezoresistive sensing is still among most important and widely used sensing mechanisms in MEMS sensing technologies due to the advantage of simple signal processing, we can take the analog signal either directly from the sensor, or directly amplify or convert to a digital signal if necessary. Compared to the piezoresistive method, the capacitive method requires a complex C-V converter as part of the ASIC and the resonant method also needs a frequency converter. As one type of pressure sensor, high-temperature piezoresistive pressure sensors are of great importance in many applications, such as industrial control, vehicle engineering and aerospace engineering [8,9,10]. Some SOI-based pressure sensors do not use an etching process but only prepare piezoresistors by ion implantation in the device layer [11]. However, piezoresistors can only work properly in environments below 330 °C [12]. To date, high-temperature absolute pressure sensors have been generally fabricated by using double-side etched SOI silicon to form the sensing elements (e.g., insulator isolated piezoresistor and pressure-sensing diaphragm) and wafer-bonding process, e.g., anodic bonding, to seal the pressure-reference cavity [13,14]. Since the anisotropic-etching-formed inclined sidewalls will occupy a large device size, the sensor chip size is quite large (e.g., 5 × 5 × 0.9 mm in [14]) and the fabrication cost cannot be very low. Therefore, it is difficult to realize miniaturization of the sensors and low-cost fabrication. Moreover, the thickness of the backside-etching formed pressure-sensing diaphragm is difficult to keep very uniform due to the non-uniformity of the used deep-Si etching, thereby resulting in sensitivity non-uniformity and low fabrication yield. Even if the etching rate could be uniform, the thickness uniformity all over the wafer still remains difficult because of the intrinsic non-uniform thickness of the original wafer before any process. Original silicon wafers normally have a thickness deviation of ±5 μm to ±20 μm, which is comparable with the desired sensing diaphragm thickness. To address the deficiency of the thickness nonuniformity of the pressure-sensing diaphragm, double-layer SOI (Si/Al_2_O_3_/Si/SiO_2_/Si) wafers, formed by wafer bonding and heteroepitaxial growth, have been used to fabricate the high-temperature pressure sensors with a rectangular diaphragm of 360 × 1140 μm^2^ and a maximum working temperature of 350 °C [15,16,17]. In the temperature range from −20 °C to 350 °C, the shift in sensitivity and offset voltage of the pressure sensor are less than −0.2% and 0.l%, respectively [16]. During the fabrication process of the double-SOI structure, a double-heteroepitaxial growth was employed to form the (100) Al_2_O_3_ for avoiding current leakage at high temperatures and the top-layer (100)-silicon for piezoresistors. The middle SiO_2_ buried layer was used as the etching self-stop layer for forming the pressure-sensing diaphragm with uniform thickness, where wet etch or dry etch from the backside of the SOI wafer can be stopped automatically. However, due to the complex fabrication process of the double-SOI wafer, the fabrication cost has to be increased. Additionally, in order to obtain a small chip size, silicon deep reactive ion etching (DRIE) has to be used to replace anisotropic wet etching to avoid the lateral size occupied by the inclined (111) etching sidewall.

To satisfy the aforementioned requirements, our previously developed MIS process [18] is employed on a (100)/(111) single-layer SOI wafer to successfully fabricate the ultra-small high-temperature (−55–350 °C) pressure sensor. Profiting from the front-sided shallow etching and self-stop wet etching, the thickness of the pressure-sensing diaphragm is uniform and controllable. Without a complex double-sided micromachining process, the wafer bonding process and cavity-SOI wafers, the chip size of the fabricated pressure sensor is as small as 0.5 × 0.5 mm. Generally, the fabricated pressure sensors exhibit quite high performance, miniaturization in chip footprint and low-cost fabrication.

## 2. Design and Modeling

**Figure 1:**
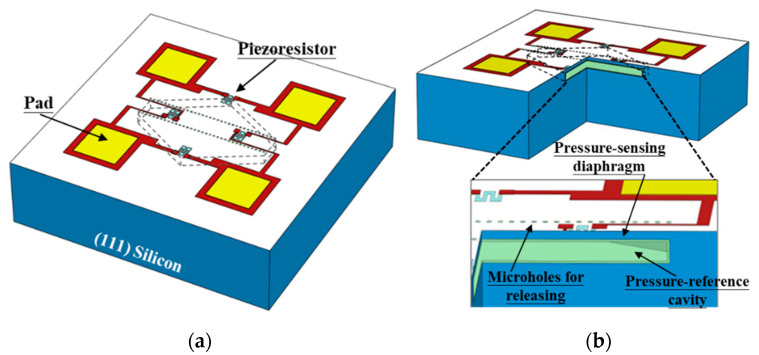
(**a**) The 3D diagram of the high-temperature pressure sensor from the single-side micromachining process; (**b**) cross-sectional view for detailing the structure of the pressure sensor.

Figure 1a shows the 3D schematic of the proposed single-side micromachined high-temperature pressure sensors. The 0.1 μm thick (100) device layer (i.e., active layer) is doped with boron ion implantation to the high level (close to solid solubility) to fabricate the piezoresistors for pressure measurement at high temperatures, thereby ensuring that the SiO_2_-layer isolated piezoresistors work stably with long-term electrical performance [12]. Here, the fabricated piezoresistors are located along a <110> orientation. The 0.14 μm thick buried oxide can effectively ensure reliable work of the sensors at very high temperatures without any leakage current effect associated with the p-n junction isolated devices [19]. Based on the special arrangement of the crystal planes in (111)-silicon wafers and the anisotropic wet-etch characteristics of single crystal silicon, taking (111) silicon as the handle layer of the hybrid SOI-silicon wafer is more beneficial to construct uniform sensing diaphragm and embedded pressure-reference cavity. As depicted in Figure 1b, after the two rows of microholes (diameter = 4 μm and the rows along the <211> orientation) are opened by using DRIE at the front side of the (111) handle layer, the sensing diaphragm can be formed by laterally excavating the silicon beneath the diaphragm by using anisotropic wet etching. Additionally, the pressure-reference cavity beneath the diaphragm is embedded into the handle layer and with six {111} etching-stop sidewalls as its structure boundary.

**Figure 2:**
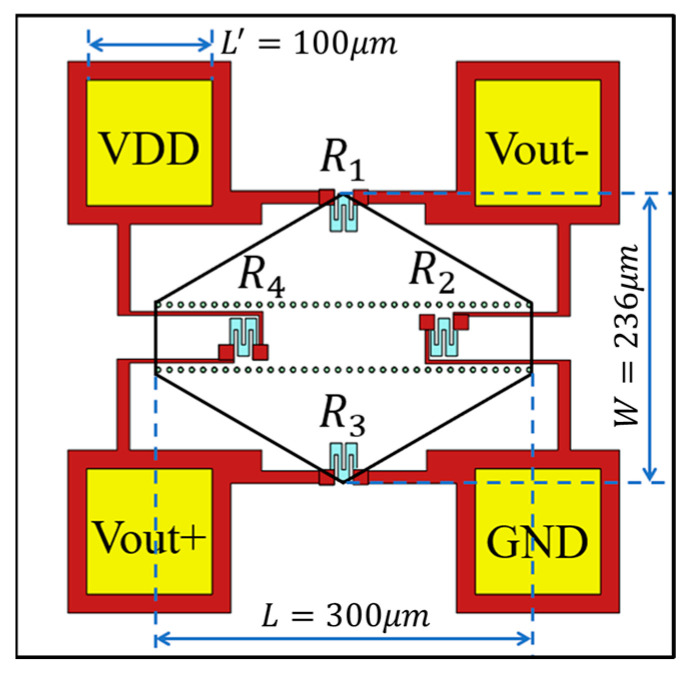
Layout of the pressure-sensing structures on the micromechanical diaphragm.

The black hexagonal solid line in Figure 2 represents the profile of our fabricated pressure-sensing diaphragm. In order to further reduce the chip size, the pads with a length $L^{\prime }$ of 100 μm are located on the place where is as close as possible to the pressure-sensing diaphragm. In addition, considering that heavily boron-doped silicon layer as trace line on the pressure-sensing diaphragm will introduce significant residual stress, and weighing the pros and cons, we use metal trace line instead of heavily boron-doped silicon layer to interconnect the piezoresistors to form a Wheatstone bridge. Attributed to the layout of the piezoresistors interconnected with uniform and symmetrical Ti/Pt/Au-trace line, the thermal hysteresis of output voltage can be effectively reduced [20].

**Figure 3:**
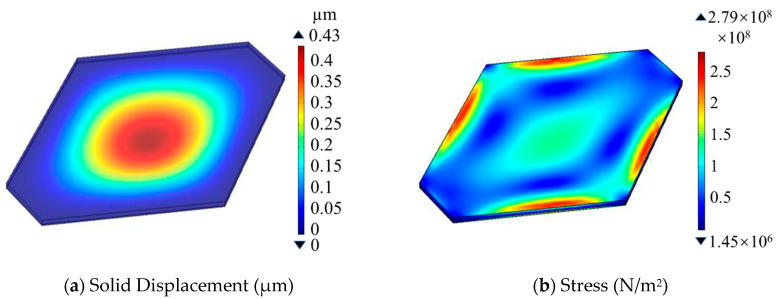

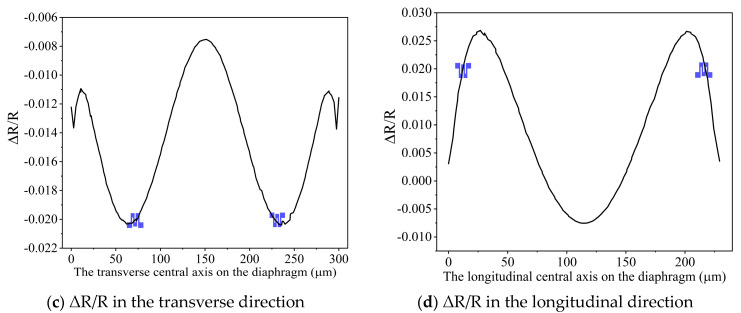
Finite-element simulation results under 1.5 MPa pressure. (**a**) Deflection distribution on the top surface of the pressure-sensing diaphragm. (**b**) Stress on the diaphragm. (**c**) ΔR/R in the transverse direction. (**d**) ΔR/R in the longitudinal direction.

After theoretic analysis according to the design rules in [21] and our previous works in [22], the dimensions of the hexagonal-shaped pressure-sensing diaphragm with the length L of 300 μm along the horizontal symmetry and the width W of 236 μm along the vertical symmetry in Figure 2 is designed, and the four Wheatstone-bridge piezoresistors are laid at the locations with maximum piezoresistive sensitivity. With applied pressure on the sensitive diaphragm, $R_{1}$ and $R_{3}$ are subjected to tensile stress, resulting in an increase in their resistance value, whereas $R_{2}$ and $R_{4}$ will decrease their resistance value. The four piezoresistors form a fully sensitive Wheatstone bridge. Additionally, a finite-element analysis by using COMSOL was implemented on the flat hexagonal-shaped pressure-sensing diaphragm with area of 42,900 μm^2^ and thickness of 8 μm to evaluate the rationality of the abovementioned design (e.g., piezoresistor arrangement and measuring range). Under an applied pressure of 1.5 MPa from the top side of the diaphragm, the COMSOL-simulated results in Figure 3a show that the maximum deflection of 0.43 μm at the center of the pressure-sensing diaphragm is far less than one fifth of the thickness of the sensitive diaphragm [23], satisfying the small deflection deformation of the sensitive diaphragm within the full range of 1.5 MPa, which is helpful in achieving a low non-linearity. As is depicted in Figure 3b, the maximum von Mises stress of $2.79\times 10^{8}$ N/m^2^ occurs at the edge of the diaphragm, which is smaller than silicon rupture stress ($3\times 10^{9}$ N/m^2^) [21], ensuring the sensor will not be damaged under 10 times of overload pressure. In addition, the rate of the stress-induced resistance change, ΔR/R, in Figure 3c,d can be derived from the stress distribution along the longitudinal direction and the transverse direction, respectively. As shown in Figure 3c,d, in order to achieve both high sensitivity and high output linearity, the four piezoresistors are placed at the locations where the transverse and longitudinal resistance relative change, ΔR/R, have the same value and ≥2%. Referring to the layout of the piezoresistors, the theoretical sensitivity of the pressure sensor is approximately 0.013 mV/V/kPa.

## 3. Sensor Fabrication

**Figure 4:**
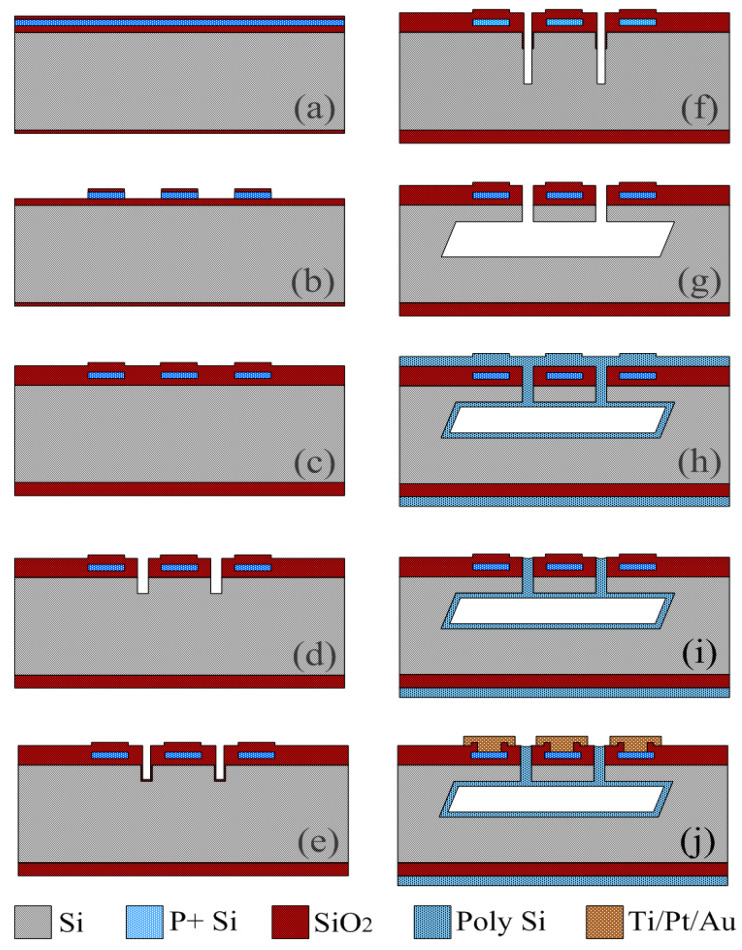
Single-sided micromachining process of the high-temperature pressure sensor.

Figure 4 details the single-side fabrication steps for the small-size high-temperature pressure sensors, and all the micromachining processes are carried out only from the front side of a single-side polished n-type (100)/(111) hybrid SOI wafers. The (100)/(111) hybrid SOI wafer with a 0.1 μm thick (100) device layer, a 0.14 μm thick buried oxide layer and a 700 μm thick (111) handle layer are prepared with a smart cut process, and the entire fabrication process is described as follows.

(a) A 0.1 μm thick SiO_2_ layer is formed by a thermal oxidation process. Additionally, the (100) device layer of the hybrid SOI silicon wafer is doped with high-dose boron ion implantation followed by a drive-in process under 1100 °C nitrogen environment for approximately 40 min. Note that the dose and energy of the boron ion implantation are 5 $e^{15}$/${\mathrm{cm}}^{2}$ and 50 keV, respectively, and the impurity concentration is controlled at the level of approximately 1.3 $e^{20}$/${\mathrm{cm}}^{3}$ after a drive-in process.

(b) p-type piezoresistors are sculptured out of the top (100) silicon layer on the buried oxide layer by using a reactive ion etch (RIE) and a deep reactive ion etch (DRIE) process sequentially.

(c) A 1.0 μm thick TEOS (tetraethyl orthosilicate) layer is deposited by a low-pressure chemical vapor deposition (LPCVD) process as the hard masking layer for the DRIE and TMAH lateral under-etching for the diaphragm release in the following.

(d) In the (111) handle layer of the hybrid SOI silicon wafer, two rows of microholes with a diameter of 4.0 μm along the <211> orientation are opened by a RIE and a DRIE process sequentially to define the thickness of the pressure-sensing diaphragm. Note that the diameter of microholes must be larger than 3 μm, otherwise the gas generated by the reaction between TMAH solution and silicon inside the substrate cannot be discharged in time due to the viscosity of TMAH solution, resulting in incomplete release of the diaphragm and the cavity at beneath.

(e) In order to protect the vertical microhole sidewalls from being etched by TMAH in the following, a 0.4 μm thick TEOS layer by a LPCVD process is deposited to cover the hole surface.

(f) The microholes in the (111) handle layer are vertically etched again by RIE to remove the TEOS layer at the bottom surface and DRIE to deepen the holes. The etching depth is equal to the height of the pressure-reference cavity for the following lateral under-etching.

(g) The SOI wafer is dipped into the anisotropic etchant of 25 wt.% TMAH at 85 °C for about 2 h to form the pressure-sensing diaphragm and the pressure-reference cavity by lateral under-etching along the <211> and <110> orientations. Additionally, then, the residual TEOS layers on the vertical sidewalls of the microholes are removed by using the buffered HF solution.

(h) The 4.0 μm thick low-stress polysilicon is deposited by a LPCVD process to seal the microholes. During the LPCVD process, the pressure in the tube furnace is merely hundreds of millitorr. After the SOI wafer is cooled down to room temperature, the sealed pressure in the cavity will become almost close to vacuum. The residual stress in the polysilicon is then eliminated after the annealing in nitrogen.

(i) The polysilicon in the front side of the SOI wafer is removed by maskless DRIE, but the polysilicon in the microholes is retained for microseals. The electric contact holes are then exposed by photolithography and RIE steps.

(j) An Ti/Pt/Au film is sputtered, patterned and sintered for the electrical interconnection of the Wheatstone bridges and the wire bonding.

**Figure 5:**
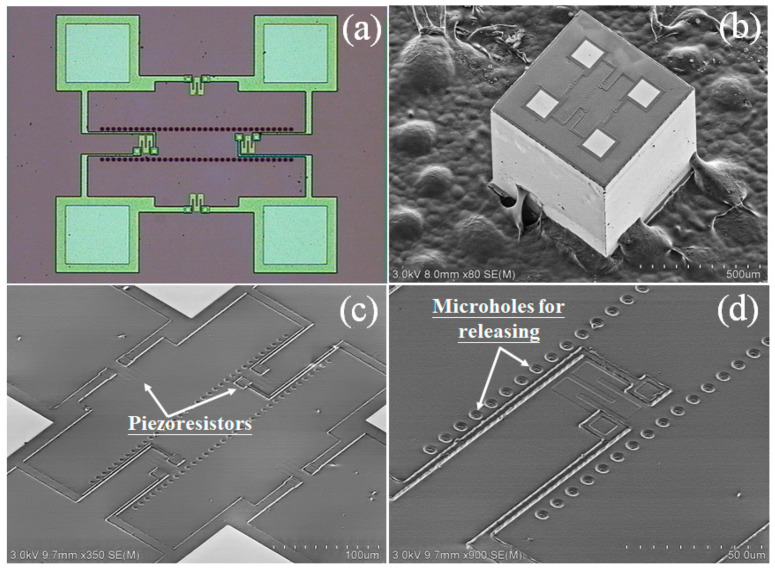
(**a**) Optical and (**b**) SEM images of the pressure sensor chip. (**c**) Magnified view of the piezoresistors; (**d**) Magnified view of the microholes that have been sealed later.

The fabricated high-temperature piezoresistive pressure sensor chip is with the images shown in Figure 5. The size of the sensor chip is as tiny as 0.5 mm by 0.5 mm. Figure 5a,b show the photograph and SEM image of the sensor chip, respectively. Figure 5c,d show the magnified views of the pressure-sensing piezoresistors and the microholes (now have been sealed by polysilicon re-filling) for lateral-etching release of the pressure-reference cavity.

## 4. Packaging and Testing

**Figure 6:**
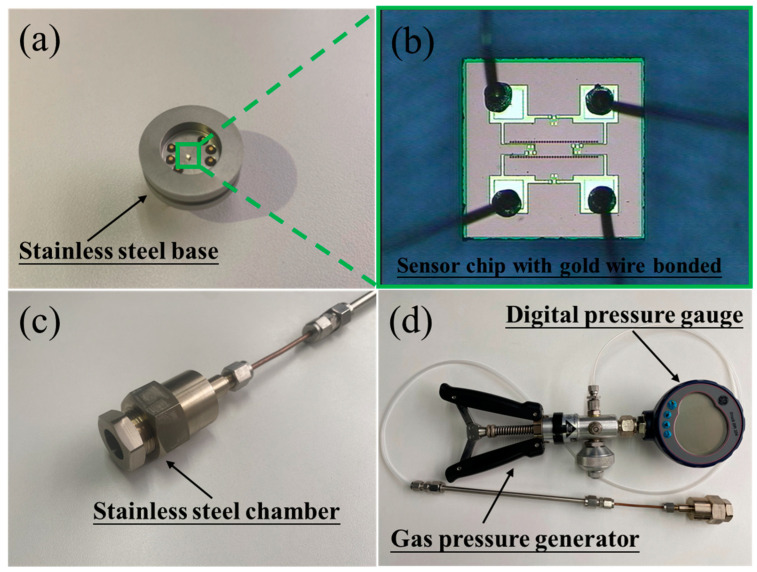
(**a**) The stainless steel base with a sensor chip fixed in it. (**b**) Magnified view of the sensor chip with gold wire bonded. (**c**) The sealed stainless steel chamber. (**d**) The system consisting of a high-accuracy digital pressure gauge and a gas pressure generator for pressure test.

After the sensor chip has been fixed inside a 316 L stainless steel base, gold wires are then used to connect the pads of the sensor chip to the pins of the base to ensure an electrical interconnection, as shown in Figure 6a,b. Then, the base is enclosed in a stainless steel chamber with a gas pipeline, which is connected to a gas pressure generator and a digital pressure gauge, as shown in Figure 6c,d.

**Figure 7:**
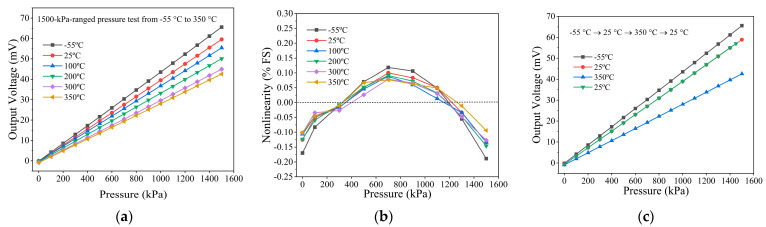
(**a**) The 1.5 MPa ranged pressure test from −55 °C to 350 °C. (**b**) The tested non-linearity and (**c**) the tested thermal hysteresis of the pressure sensor within the temperature range from −55 °C to 350 °C.

The fabricated high-temperature pressure sensors are powered by a DC voltage of 3.3 V. The following testing results are obtained without signal amplification and compensation for temperature drift used. The measurement is within the absolute pressure range of 0~1500 kPa, where the reference pressure is vacuum. At various temperature from −55 °C to 350 °C, Figure 7a shows the tested output voltage of the sensor in terms of applied pressure. At a room temperature of 25 °C, the pressure sensor exhibits a full-scale output voltage of 59.55 mV/1500 kPa/3.3 VDC, i.e., the sensitivity is 0.012 mV/V/kPa. Within the temperature range of −55~350 °C, the overall accuracy (combining hysteresis, non-linearity and repeatability) is evaluated as 0.17% FS, with the non-linearity error always being smaller than ±0.19% FS that can be seen in Figure 7b and Appendix A. In the same temperature, the drifts of output voltage caused by the residual stress on the wire trace under thermal cycle loading will influence the performance of the high-temperature pressure sensor, and it is called the thermal hysteresis phenomenon [20]. Our fabricated sensor has a low thermal hysteresis of 0.15%·FS within the whole temperature range, as shown in Figure 7c and Appendix B.

**Figure 8:**
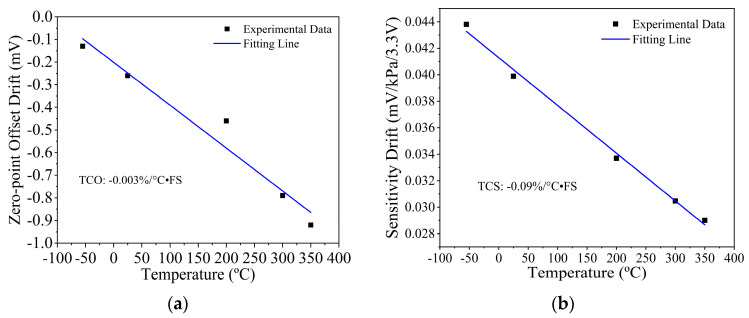
(**a**) Tested zero-point temperature drift and (**b**) sensitivity temperature drift within the whole temperature range of −55 °C to 350 °C.

As shown in Figure 8a, the tested temperature coefficient of offset (TCO) of the 1.5 MPa pressure sensor is as low as −0.003%/°C·FS. Additionally, the testing results of temperature coefficient of sensitivity (TCS) shown in Figure 8b is −0.09%/°C·FS for the whole temperature range.

In addition, the impact of high- and low-temperature cycling on the long-term stability of sensors will also be evaluated. Herein, high-temperature (operating for 2 h at 350 °C) and low-temperature (operating for 2 h at −55 °C) cycle continuously affects the fabricated sensor for seven days. We find that the time zero drift is less than 0.12% FS and no time sensitivity drift is observed.

Table 1 shows the performance comparison with other pressure sensors, which confirms that our fabricated ultra-small pressure sensors exhibit good performance at high temperatures.

## 5. Conclusions

By combining the single-layer (100)/(111) SOI silicon wafer and a MIS process for single-sided micromachining that our group developed, a high-performance high-temperature piezoresistive pressure sensor was designed, fabricated and tested. The pressure sensor features a small chip size and high-yield low-cost batch production potential. Benefitting from the optimal design of the sensor structure, the pressure sensor exhibits good performance within the temperature range from −55 °C to 350 °C and is promising in aerospace and industrial applications.

## Figures and Tables

**Table 1:** Performance comparison of pressure sensors.

Sensor	Area (mm^2^)	Temperature Range (°C)	Overall Accuracy (%FS)	TCO (%°C·FS)	TCS (%°C·FS)
Sensor in [23]	4	20~220	±0.5	/	/
Sensor in [24]	/	20~220	/	−0.06	−0.13
Sensor in [25]	Not less than 12.96	−20~150	0.34	1.8	−0.15
Our work	0.25	−55~350	0.17	−0.003	−0.09

## Appendix A

The non-linearity of a pressure sensor indicates the extent to which the curve of the relationship between the output and the input of the sensor deviates from the selected operating curve and can be expressed by the following equation:

$$Nonlinearity=\frac{\Delta V_{max}}{V_{FS}}\times 100\%$$

where $\Delta V_{max}$ is the maximum deviation between the output voltage of the pressure sensor and the linear fit value. $V_{FS}$ is the full-scale output of the pressure sensor.

**Table A1:** Non-linearity of the pressure sensor.

	Temperature (°C)	−55	25	100	200	300	350
Pressure (kPa)							
0		−0.17022	−0.12524	−0.10624	−0.12367	−0.10237	−0.09993
100		−0.08267	−0.05019	−0.04604	−0.05929	−0.03393	−0.04557
300		−0.00936	−0.01661	−0.01561	−0.00728	−0.02621	−0.01098
500		0.069758	0.050221	0.045523	0.052056	0.026293	0.067269
700		0.118444	0.100343	0.088672	0.091612	0.07879	0.076475
900		0.106272	0.083635	0.059906	0.071834	0.065666	0.062666
1100		0.048458	0.050221	0.013161	0.032278	0.030667	0.048858
1300		−0.055	−0.03331	−0.03358	−0.04683	−0.04808	−0.01098
1500		−0.18889	−0.13356	−0.13427	−0.14572	−0.12683	−0.09383

## Appendix B

The thermal hysteresis of a pressure sensor is the extent to which its output voltage deviates when it passes through the same temperature point within a certain temperature range and can be expressed by the following equation:

$$Thermal\;hysteresis=\frac{\Delta V{’}_{max}}{V{’}_{FS}}\times 100\%$$

where $\Delta V{’}_{max}$ is the maximum deviation of the output voltage of the pressure sensor at the same temperature point of a temperature rise and fall cycle. $V{’}_{FS}$ is the full-scale output of the pressure sensor at 25 °C.

**Table A2:** Thermal hysteresis of the pressure sensor.

	Pressure (kPa)	0	100	200	300	400	500	600	700
Temperature (°C)									
−55		−0.14	4.23	8.57	12.9	17.25	21.61	25.97	30.34
25		−0.63	3.34	7.27	11.2	15.15	19.1	23.05	27.02
350		−0.88	2.02	4.89	7.77	10.65	13.53	16.43	19.32
25		−0.62	3.35	7.29	11.23	15.18	19.14	23.09	27.06
	**Pressure (kPa)**	**800**	**900**	**1000**	**1100**	**1200**	**1300**	**1400**	**1500**
**Temperature (°C)**									
−55		34.73	39.11	43.51	47.91	52.32	56.74	61.17	65.59
25		30.98	34.96	38.94	42.92	46.91	50.91	54.91	58.92
350		22.22	25.12	28.02	30.92	33.83	36.74	39.66	42.57
25		31.04	35.02	39	43	47	50.99	55	59.01

## References

[1] Smith C.S. (1954). Piezoresistance Effect in Germanium and Silicon. Phys. Rev..

[2] Li X., Lu D. (1999). A micromachined piezoresistive angular rate sensor with a composite beam structure. Sens. Actuators A Phys..

[3] Wang J., Li X. (2010). A high-performance dual-cantilever high-shock accelerometer single sided micromachined in (111) silicon wafers. J. Microelectromech. Syst..

[4] Yang H., Lu D. (1998). A pressure transducer with a single-sided multilevel structure by maskless etching technology. Mechatronics.

[5] Bock W.J., Eftimov T., Molinar G.F., Wisniewski R. Free active element bulk-modulus high-pressure transducer based on fiber-optic displacement sensor. Proceedings of the IEEE Instrumentation and Measurement Technology Conference.

[6] Morten B., de Cicco G., Prudenziati M. (1992). Resonant pressure sensor based on piezoelectric properties of ferroelectric thick films. Sens. Actuators A Phys..

[7] Mitrakos V., Macintyre L., Denison F., Hands P., Desmulliez M. (2017). Design, manufacture and testing of capacitive pressure sensors for low-pressure measurement ranges. Micromachines.

[8] Eaton W., Smith J. (1997). Micromachined pressure sensors: Review and recent developments. Smart Mater. Struct..

[9] Fleming W. (2001). Overview of automotive sensors. IEEE Sens. J..

[10] Fiorillo A.S., Critello C.D., Pullano S.A. (2018). Theory, technology and applications of piezoresistive sensors: A review. Sens. Actuators A Phys..

[11] Xu Z., Yan J., Ji M., Zhou Y., Wang D. (2022). An SOI-Structured Piezoresistive Differential Pressure Sensor with High Performance. Micromachines.

[12] Guo S., Eriksen H., Childress K., Fink A., Hoffman M. (2009). High Temperature Smart-Cut SOI Pressure Sensor. Sens. Actuators A Phys..

[13] Li S., Liang T., Wang W., Hong Y., Zheng T., Xiong J. (2015). A Novel SOI Pressure Sensor for High Temperature Application. J. Semicond..

[14] Meng Q., Lu Y., Wang J., Chen D., Chen J. (2021). A piezoresistive pressure sensor with optimized positions and thickness of piezoresistors. Micromachines.

[15] Chung G. (1990). Novel pressure sensors with multilayer SOI structures. Electron. Lett..

[16] Chung G., Suzaki T. (1991). High-performance pressure sensors using double siliconon-insulator structures. Rev. Sci. Instrum..

[17] Chung G. (1993). Thin SOI structures for sensing and integrated circuit applications. Sens. Actuators A Phys..

[18] Wang J., Li X. (2013). Package-friendly piezoresistive pressure sensors with on-chip integrated packaging-stress-suppressed suspension (PS3) technology. J. Micromech. Microeng..

[19] Anthony D., Alan H. Ultra high temperature, miniature, SOI sensors for extreme environments. Proceedings of the IMAPS International HiTEC 2004 Conference.

[20] Chiang H., Chiang K. Investigation of the hysteresis phenomenon of a silicon-based piezoresistive pressure sensor. Proceedings of the 2007 International Microsystems, Packaging, Assembly and Circuits Technology.

[21] Bao M. (2005). Analysis and Design Principles of MEMS Devices.

[22] Wang J.C., Xia X.Y., Li X.X. (2012). Monolithic integration of pressure plus acceleration composite TPMS sensor with a single-Sided micromachining technology. IEEE/ASME J. Microelectromech. Syst..

[23] Yao Z. (2016). A High-Temperature Piezoresistive Pressure Sensor with an Integrated Signal-Conditioning Circuit. Sensors.

[24] Yao Z. (2016). Passive Resistor Temperature Compensation for a High-Temperature Piezoresistive Pressure Sensor. Sensors.

[25] Li C., Cordovilla F., Jagdheesh R., Ocaña J.L. (2018). Design Optimization and Fabrication of a Novel Structural SOI Piezoresistive Pressure Sensor with High Accuracy. Sensors.

